# The impact of psychosocial variables on initial presentation and surgical outcome for ulnar-sided wrist pathology: a cohort study with 1-year follow-up

**DOI:** 10.1186/s12891-022-05045-x

**Published:** 2022-02-01

**Authors:** J. S. Teunissen, M. J. W. van der Oest, D. E. van Groeninghen, R. Feitz, S. E. R. Hovius, E. P. A. Van der Heijden

**Affiliations:** 1 Department of Plastic, Reconstructive and Hand Surgery, Radboud University Medical Centre, Radboud Institute for Health Sciences, Geert Grooteplein Zuid 10, 6525 GA Nijmegen, The Netherlands; 2 Hand and Wrist Centre, Xpert Clinics, Laarderhoogtweg 12, 1101 EA Amsterdam, The Netherlands; 3 Department of Plastic, Reconstructive and Hand Surgery, Erasmus MC, University Medical Centre Rotterdam, Doctor Molewaterplein 40, 3015 GD Rotterdam, The Netherlands; 4 Department of Rehabilitation Medicine, Erasmus MC, University Medical Centre Rotterdam, Doctor Molewaterplein 40, 3015 GD Rotterdam, The Netherlands; 5 Department of Plastic Surgery, Jeroen Bosch Ziekenhuis, Henri Dunantstraat 1, 5223 GZ ‘s-Hertogenbosch, The Netherlands

**Keywords:** Ulnar sided wrist pain, PRWHE, Illness perception, Pain catastrophising, Depression

## Abstract

**Aims:**

Ulnar-sided wrist pain has historically been equated to lower-back pain of wrist surgery. Little is known about the relationship between psychosocial profile and the manifestation of ulnar-sided wrist pathology and their treatment outcomes. This study aimed to determine the impact of pain catastrophising, psychological distress, illness perception, and patients’ outcome expectations on patient-reported pain and hand function before and one year after surgery for ulnar-sided wrist pathology.

**Patients and Methods:**

We included patients who underwent surgical treatment for ulnar-sided wrist pathology. Before surgery, patients completed the Pain Catastrophising Scale (PCS), Patient Health Questionnaire (PHQ), Brief-Illness Perception Questionnaire (B-IPQ), and Credibility/Expectancy Questionnaire (CEQ). Pain and dysfunction were assessed before (n = 423) and one year after surgery (n = 253) using the Patient Rated Wrist/Hand Evaluation (PRWHE). Hierarchical linear regression was used to assess the relationship between psychosocial factors and the preoperative PRWHE score, postoperative PRWHE score, and change in PRWHE.

**Results:**

Psychosocial variables explained an additional 35% of the variance in preoperative PRWHE scores and 18% on postoperative scores. A more negative psychosocial profile was associated with higher (worse) preoperative PRWHE scores (PCS: B = 0.19, CI = [0.02–0.36]; B-IPQ Consequences: B = 3.26, CI = 2.36–4.15; and B-IPQ Identity, B = 1.88 [1.09–2.67]) and postoperative PRWHE scores (PCS: B = 0.44, CI = [0.08–0.81]) but not with the change in PRWHE after surgery. Higher treatment expectations were associated with a lower (better) postoperative PRWHE score (CEQ expectancy: B = -1.63, CI = [-2.43;-0.83]) and a larger change in PRWHE scores (B =|1.62|, CI = [|0.77; 2.47|]).

**Conclusion:**

A more negative psychosocial profile was associated with higher pain levels and dysfunction preoperatively and postoperatively. However, these patients showed similar improvement as patients with a more feasible psychosocial profile. Therefore, patients should not be withheld from surgical treatment based on their preoperative psychosocial profile alone. Boosting treatment expectations might further improve treatment outcomes.

**Level of evidence:**

III (Cohort study).

**Supplementary Information:**

The online version contains supplementary material available at 10.1186/s12891-022-05045-x.

## Introduction

Chronic conditions of the wrist can be challenging to manage. Especially ulnar-sided wrist pain is playfully equated to the “lower back pain” or “black-box” of the wrist due to the diverse nature of chronic complaints, insidious appearance and resulting frustration as well as the anatomical complexity [1–4].

The anatomy of the ulnar-sided wrist, the diagnosis and treatment options have recently been summarised in a comprehensive review [4]. In short, treatment often starts with nonoperative modalities, including anti-inflammatory drugs, splinting, corticoid steroid injection, and hand therapy [4]. Subsequent surgical treatment may be needed to reduce symptoms further and improve function. The Four-Leaf Clover treatment algorithm proposed by Kakar and Garcia-Elias recommends surgical treatment based on the status of 4 main structures related to ulnar-sided wrist pain [5]: A) bone deformity (e.g. ulnar impaction syndrome), B) cartilage defects (e.g. distal radioulnar joint osteoarthritis and pisotriquetral osteoarthritis), C) TFCC injury, and D) unstable Extensor Carpi Ulnaris. Treatment should mainly be directed to the type of pathological structure(s) focussing on the reconstruction of the anatomy by A) corrective osteotomy (e.g. ulnar shortening osteotomy), B) DRUJ arthroplasty (e.g. u-head) / Pisiformectomy, C) ligament reconstruction (e.g. Adams or TFCC reinsertion), and D) ECU stabilisation.

While effort has been put into understanding ulnar-sided wrist pathology based on the anatomy and biomechanics [1, 4, 5], psychosocial factors (e.g. pain catastrophising, anxiety and depression, and illness perception) have been scarcely investigated in these patients. However, this may be equally important as previous studies have shown that anatomical findings during diagnostic workup only partly relate to the amount of ulnar-sided wrist pain [6–8].

The relationship between psychosocial profile and patient-reported pain and dysfunction is becoming well recognised for common musculoskeletal pathology. For example, pain catastrophising and depression were associated with higher scores of pain and dysfunction at presentation in patients with hip [9], thumb [10], or spine pathology [11]. Furthermore, illness perception was also associated with higher pain and dysfunction in patients with Quervain’s tenosynovitis [12], thumb base osteoarthritis [10], and carpal tunnel syndrome [13]. Some studies found that the patients’ psychosocial profile was even more associated with their pain and dysfunction than the severity of their pathology [10, 14].

Next to the potentially better understanding of patient-reported pain and dysfunction in patients with ulnar-sided wrist pathology, psychosocial factors may be determinants for the outcome of surgery. A meta-analysis on the outcome of total knee replacement reported that a more negative psychosocial profile was associated with worse outcomes [15]. However, other studies on spinal surgery or carpal tunnel release found that a more negative preoperative psychosocial profile did not compromise the outcome of surgery [11, 16].

The association between psychosocial variables and patient-reported pain and dysfunction have been scarcely investigated in patients with ulnar-sided wrist pathology. Also, their effect on the outcome of surgery is unclear. Therefore, this study aimed to determine the impact of pain catastrophising, psychological distress, illness perception, and patients’ outcome expectations on patient-reported pain and hand function both before and at one year after surgery for ulnar-sided wrist pathology.

## Patients and Methods

### Study design and setting

We studied prospectively gathered data on a consecutive cohort of patients that underwent surgical treatment of ulnar-sided wrist pathology between September 2017 and June 2020 at Xpert Clinics, The Netherlands. All surgeons at our institution are certified by the Federation of European Societies for Surgery of the Hand and/or fellowship trained.

After their first consultation with a hand surgeon, all our patients were invited to be included in the Hand and Wrist Cohort, a routine outcome measurement system. Upon agreement, they received secure web-based questionnaires using GemsTracker [17]. Three reminders were sent to the patients for each round of questionnaires. The exact research setting of our study group has been reported previously [18].

We report this study using the Strengthening the Reporting of Observational Studies in Epidemiology (STROBE) statement [19]. International Review Board approval was obtained from the ethics committee of the Erasmus Medical Center Rotterdam (NL/sl/MEC-2018–1088). All patients provided written informed consent for their data to be anonymously used in this study.

### Participants

We identified adult patients in the Hand and Wrist Cohort scheduled for surgical treatment for ulnar-sided wrist pathology by the treatment codes: USO; TFCC reinsertion; pisiformectomy; u-head implant; and Adams procedure. Generally, conservative treatment was initiated by a short period of immobilisation, followed by a rigorous program of wrist exercises. After careful anamnesis, physical examination, and imaging (e.g. MRI, CT, or wrist arthroscopy), surgical treatment was considered if symptoms persisted for more than three months or if patients did not want further nonoperative management. Surgical treatment was directed to the type of pathological structure(s) focussing on the reconstruction of the anatomy [5]. Exclusion criteria for this study were: 1) patients younger than 18 years; 2) patients with incomplete demographic and psychosocial data; 3) patients who underwent surgical procedures that were less prevalent than 30 in the dataset after applying exclusion criteria 1 and 2.

### Baseline demographics

After the first consultation with a hand surgeon, sociodemographic characteristics were routinely collected. The variables evaluated in this study were age, sex, type of work, symptom duration, and whether the dominant side was affected.

### Psychosocial variables

The Pain Catastrophising Scale(PCS) was used to measure pain catastrophising [20, 21]. We evaluated the PCS total score, ranging from 0–52 (0 = no catastrophising behaviour; 52 = severe catastrophising behaviour). A PCS total score ≥ 30 is considered a clinically relevant level of catastrophising.

The Patient Health Questionnaire-4 (PHQ), a combination of the PHQ-2 and Generalised Anxiety Disorder (GAD)-2, measured psychological distress (anxiety and depression) [22]. We evaluated the PHQ total score, ranging from 0–12 (0 = no psychological distress; 12 = severe psychological distress). A PHQ total score of 6–8 is considered a “yellow flag”, and ≥ 9 a “red flag”.

The Brief Illness Perception Questionnaire(B-IPQ) was used to measure how patients perceive their illness [23, 24]. As recommended [23], we evaluated the subscales (range 0–10) separately. For the items Personal control (how much control patients feel they have over their illness), Treatment control (how much patients think their treatment will help their illness), and Comprehension (how well patients understand their illness), a higher score is better. For all other items: Consequences (how much the illness affects the patients’ life), Timeline (how long patients expect their illness to last), Identity (how much patients contribute symptoms to their hand condition), Concern (How concerned patients are), and Emotion (how much the patients are emotionally affected by the illness) a lower score is better. The Credibility/Expectations Questionnaire (CEQ) measured the patients’ expectations and credibility of the treatment outcomes [25]. It consists of 6 questions, and the scores range from 3–27 (3 = low expectations and credibility; 27 = high expectations and credibility). In this study, we only evaluated the Expectancy subscale. Due to collinearity concerns, the Treament control subscale was not evaluated. We used the validated Dutch language versions (DLV) for all questionnaires [21, 24, 26, 27].

### Outcome measure

The Patient Rated Wrist/Hand Evaluation-DLV (PRWHE) measured patient-reported pain and dysfunction before surgery and one year after surgery [28, 29]. The 1-year follow-up duration was chosen based on the alignment of clinical follow-up, the pathophysiology of the condition and expected treatment effect [18]. This was in line with international recommendations [30]. We evaluated the PRWHE total score, ranging from 0–100 (0 = no pain and dysfunction; 100 = severe pain and dysfunction). Outcomes from some of the included patients in this study have been evaluated before [31, 32].

### Sample size

For a fixed regression model with an R^2^ deviation from zero with an effect size F^2^ of 0.10, α of 0.05, 16 predictors and a sample size of 423, we reached a power of 99,5%. For the model at follow-up with the same effect size, α, 18 predictors, and 253 patients, we reached a power of 87,9%

### Statistical analysis

We checked continuous data for normal distributions with histograms and quantile–quantile plots. Normally distributed data were displayed as mean values with standard deviations (SD), and skewed data were displayed as mean values with inter-quartile ranges (IQR).

We performed a hierarchical multivariable linear regression to investigate the relative contribution of different variables to the explained variance in the amount of pain and dysfunction before surgery (PRWHE total score; model 1) and after surgery (model 2). For model 1, we consecutively added sociodemographics, scheduled surgical procedure, PCS + PHQ scores, and B-IPQ scores. For model 2, we also stepwise added the CEQ expectation subscale and the preoperative PRHWE score. For model 3, the outcome was the change score in PRWHE before and after surgery. Similar to other studies, PCS and PHQ scores were added simultaneously but separately from B-IPQ since pain catastrophising and psychological distress have been studied more extensively [10, 12, 13]. Outcome expectations were only evaluated in the outcome models (model 2) [33]. The variable treatment was added in the models as an instrumental variable for diagnosis/pathology.

Regression coefficients (B) with corresponding 95% confidence intervals and standardised coefficients (β) are reported for all variables. To illustrate the explained variance of different models, R^2^, adjusted R^2^, and significance of F change are reported per step. All linear regression model assumptions were checked and satisfied.

We calculated Pearson correlation coefficients and variance inflation factors (VIF) to investigate whether the correlation between psychosocial variables did not bias our estimates. We interpreted the Pearson correlation coefficients as suggested by Hinkle et al. [34] and VIF by Gareth [35].

Because data were collected during daily clinical practice, and participation in the routine outcome measurement was voluntary, missing data were expected. We tested for significant differences in demographic characteristics and, when available, preoperative questionnaire scores between patients who completed all questionnaires (complete responders) versus patients who completed none or only some (non/partial responders) using analysis of variance or Chi^2^ tests.

The sample size was calculated using GPower 3.1 [36]. All other analyses were performed in software package R, version 3.6.1. For all tests, a p-value smaller than 0.05 was considered statistically significant.

## Results

A total of 603 patients with ulnar-sided wrist pathology were scheduled for surgical treatment during the study period. We excluded eight patients younger than 18 years, one patient with incomplete demographic data, and 12 patients who underwent a surgical procedure less prevalent than 30 times in the dataset.

**Fig. 1:**
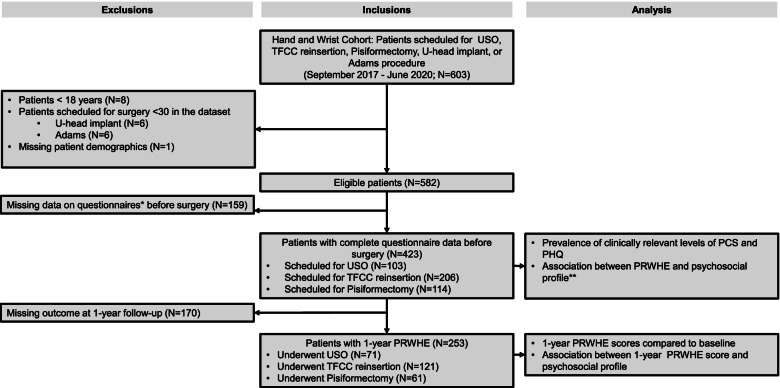
Flowchart of the study. Abbreviations: USO = Ulnar Shortening Osteotomy; TFCC = Triangular Fibrocartilaginous Complex; PRWHE = Patient Rated Wrist/Hand Evaluation. *PCS = Pain Catastrophising Scale; PHQ = Patient Health Questionnaire; B- IPQ = Brief-Illness Perception Questionnaire; CEQ = Credibility Expectancy Questionnaire. **PCS, PHQ, B-IPQ

Of the 582 eligible patients, 423 (initial response rate: 73%) completed all questionnaires before surgery (103 USO, 206 TFCC reinsertion, 114 pisiformectomy) and were enrolled in the study. Of these patients, 253 (retention rate is 60%) also completed the follow-up assessment. This subgroup of patients was used for models 2 and 3 (Fig. 1).

### Patient characteristics

The sociodemographic characteristics, treatment, and psychosocial scores of the 423 included patients are displayed in Table 1. The mean age was 44 (SD 14), and 25% of the patients were males.

**Table 1:** Study population characteristics. Values indicate means with standard deviations unless stated otherwise

Characteristic	All	Complete baseline	Complete baseline + 1-year PRWHE	*P*-value*	Range of possible values
N	582	423	253		
Age (years)	43 (15)	44 (14)	45 (14)	0.305	
Sex = Females, N (%)	405 (70)	316 (75)	202 (80)	*0.007*	
Duration of symptoms (mos.), median [IQR]	12 [6, 24]	12 [6, 18]	12 [6, 18]	0.626	
Type of work, N (%)				0.997	
None	118 (20)	83 (20)	54 (21)		
Light	182 (31)	136 (32)	77 (30)		
Medium	182 (31)	135 (32)	81 (32)		
Heavy	100 (17)	69 (16)	41 (16)		
Dominant side affected = No, N (%)	255 (44)	182 (43)	111 (44)	0.963	
Second opinion = No, N (%)	515 (88)	373 (88)	225 (89)	0.957	
Treatment, N (%)				0.742	
Ulnar shortening osteotomy	144 (25)	103 (24)	71 (28)		
TFCC reinsertion	294 (51)	206 (49)	121 (48)		
Pisiformectomy	144 (25)	114 (27)	61 (24)		
Preoperative PRWHE total score	63 (18)	63 (17)	65 (17)	0.495	0–100
Postoperative PRWHE total score	32 (24)	NaN (NA)	30 (24)	0.489	0–100
PCS score	13 (10)	13 (10)	13 (10)	0.997	0–52
PHQ score	2 (3)	2 (3)	2 (3)	0.980	0–12
B-IPQ Consequences	8 (2)	7 (2)	7 (2)	0.906	0–10
B-IPQ Timeline	6 (2)	6 (2)	6 (2)	0.307	0–10
B-IPQ Personal Control	4 (2)	4 (2)	4 (2)	0.975	0–10
B-IPQ Identity	7 (2)	7 (2)	7 (2)	0.794	0–10
B-IPQ Concern	6 (2)	6 (2)	6 (2)	0.988	0–10
B-IPQ Understanding	8 (2)	8 (2)	8 (2)	0.973	0–10
B-IPQ Emotional Respons	5 (3)	5 (3)	5 (3)	0.972	0–10
CEQ Expectancy	22 (4)	22 (4)	22 (3)	0.404	3–27

*SD* standard deviation, *IQR* interquartile range, *TFCC* Triangular Fibrocartilaginous Complex, *PRWHE* Patient Rated Wrist/Hand Evaluation, *PCS Pain Catastrophising Scale, *PHQ* Patient Health Questionnaire, *B- IPQ* Brief-Illness Perception Questionnaire, *CEQ* Credibility Expectancy Questionnaire. *P-values indicates the difference between the three groups

Clinically significant levels of pain catastrophizing were found in 7% of the patients and psychological distress in 10%. There was no difference in the prevalence of abnormal levels of pain catastrophizing (USO: 7%; TFFC reinsertion: 7%; Pisiformectomy: 8%; P = 0.952) and psychological distress (USO: 12%; TFFC reinsertion: 10%; Pisiformectomy: 9%; P = 0.770) based on treatment.

Non/partial responders more often males than complete responders (P = 0.007). There were no differences between these groups in other demographics or PRWHE, PCS, PHQ, IPQ, and CEQ scores (Table 1).

### Association between psychosocial variables and preoperative pain and dysfunction

**Fig. 2:**
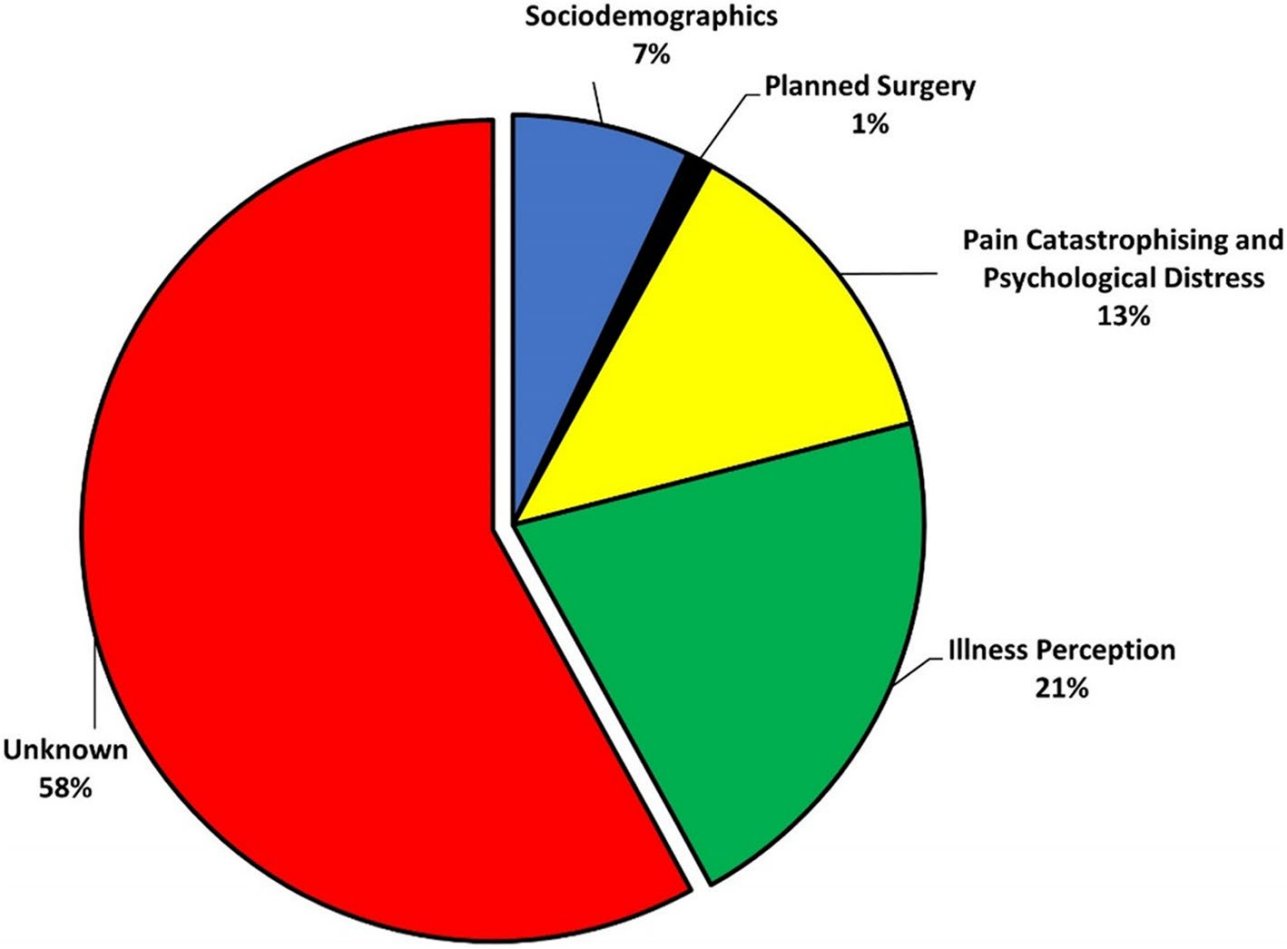
Pie chart on the increase in explained variance (R^2^) of preoperative pain and function measured with the Patient Rated Wrist/Hand Evaluation (PRWHE). Each slice represents the added R^2^ from a set of variables consecutively added to the linear regression models

The psychosocial factors increased the explained variance of preoperative PRHWE total score from 7% (sociodemographics and scheduled treatment only) to 42% (Additional file 2; Fig. 2). Female sex (B = 4.92; *β* = 0.12), higher age (B = 0.18; *β* = 0.15), higher PCS score (B = 0.19; *β* = 0.11), higher B-IPQ Consequence score (B = 3.26; *β* = 0.36), and higher B-IPQ Identity (B = 1.88; *β* = 0.23) were independently associated with higher (worse) preoperative PRWHE total score (Table 2).

**Table 2:** Results from the final step of the multivariable linear regression model on the Patient Rated Wrist/Hand Evaluation (PRWHE) before surgery, one year after surgery, and the change score. Unadjusted beta’s (B) with 95% confidence intervals (CI) and adjusted beta’s (β) are reported

	**Preop. PRWHE**		**1-year postop. PRWHE**		**Change in PRWHE**	
**Variable**	**B [95%CI]**	**Β**	**B [95%CI]**	**Β**	**B [95%CI]**	**Β**
Sex = Females	4.92 [ 1.79; 8.05]^c^	0,12	-1.46 [ -8.42; 5.49]	-0,02	4.87 [ -12.2; 2.47]	-0.08
Age (yrs.)	0.18 [ 0.07; 0.28]^b^	0,15	0.02 [ -0.21; 0.25]	0,01	-0.06 [ -0.3; 0.18]	-0.04
Dominant side affected = No	1.89 [ -0.78; 4.56]	0,05	3.78 [ -1.72; 9.29]	0,08	2.64 [ -3.24; 8.52]	0.06
Type of work = Light (ref = none)	2.33 [ -1.51; 6.17]	0,06	-4.23 [ -12.18; 3.71]	-0,08	-5.57 [ -14.06; 2.92]	-0.11
Type of work = Medium (ref = none)	3.68 [ -0.18; 7.54]	0,1	0.83 [ -7.11; 8.76]	0,02	-0.39 [ -8.87; 8.08]	-0.01
Type of work = Heavy (ref = none)	2.24 [ -2.32; 6.8]	0,05	-5.29 [ -14.76; 4.18]	-0,08	-5.83 [ -15.96; 4.31]	-0.09
Second opinion = No	-2.72 [ -6.93; 1.49]	-0,05	-2.36 [ -11.45; 6.73]	-0,03	0 [ -9.69; 9.69]	0.00
Duration of symptoms (mos.)	0 [ -0.05; 0.05]	0	0.06 [ -0.1; 0.22]	0,05	0.04 [ -0.13; 0.22]	0.03
Treatment = TFCC reinsertion (ref = USO)	-1.05 [ -4.49; 2.38]	-0,03	0.66 [ -6.44; 7.76]	0,01	1.67 [ -5.92; 9.25]	0.04
Treatment = Pisiformectomy (ref = USO)	-0.85 [ -4.56; 2.86]	-0,02	-3.22 [ -10.74; 4.3]	-0,06	-2.08 [ -10.11; 5.96]	-0.04
PCS score	0.19 [ 0.02; 0.36]^a^	0,11	0.44 [ 0.08; 0.81]^a^	0,19	0.33 [ -0.06; 0.72]	0.14
PHQ score	0.3 [ -0.31; 0.9]	0,04	-0.02 [ -1.29; 1.26]	0	-0.32 [ -1.68; 1.04]	-0.04
B-IPQ Consequences	3.26 [ 2.36; 4.15]^c^	0,36	0.31 [ -1.73; 2.35]	0,02	-2.15 [ -4.15; -0.15]^a^	-0.17
B-IPQ Timeline	0.09 [ -0.55; 0.73]	0,01	-0.33 [ -1.77; 1.1]	-0,03	-0.22 [ -1.75; 1.32]	-0.02
B-IPQ Personal Control	-0.23 [ -0.83; 0.37]	-0,03	0.27 [ -1; 1.55]	0,02	0.5 [ -0.86; 1.86]	0.05
B-IPQ Identity	1.88 [ 1.09; 2.67]^c^	0,23	0.53 [ -1.23; 2.3]	0,04	-0.61 [ -2.45; 1.23]	-0.05
B-IPQ Concern	-0.62 [ -1.32; 0.08]	-0,09	-1.19 [ -2.66; 0.29]	-0,12	-0.92 [ -2.5; 0.66]	-0.10
B-IPQ Understanding	0.04 [ -0.67; 0.74]	0	0.19 [ -1.23; 1.61]	0,02	0.18 [ -1.34; 1.69]	0.02
B-IPQ Emotional Respons	0.58 [ -0.09; 1.25]	0,09	1.08 [ -0.33; 2.49]	0,13	0.84 [ -0.67; 2.35]	0.10
CEQ Expectancy	NA	NA	-1.63 [ -2.43; -0.83]^c^	-0.25	-1.62 [ -2.47; -0.77]^c^	-0.25
Preop PRWHE total score	NA	NA	0.36 [ 0.15; 0.57]^b^	0.25	NA	NA
R^2^	0.42		0.26		0.14	
Adjusted R^2^	0.39		0.20		0.07	

*SD* standard deviation, *IQR* interquartile range, *TFCC* Triangular Fibrocartilaginous Complex, *PRWHE* Patient Rated Wrist/Hand Evaluation, *PCS Pain Catastrophising Scale, *PHQ* Patient Health Questionnaire, *B- IPQ* Brief-Illness Perception Questionnaire, *CEQ* Credibility Expectancy Questionnaire. ^a^p < 0.05; ^b^p < 0.01; ^c^p < 0.001

### Effect of psychosocial variables on postoperative pain and dysfunction

Mean PRWHE scores improved after treatment, irrespective of the type of surgery (each P < 0.001; Table 3).

**Table 3:** Mean (SD) preoperative and 1-year postoperative Patient-Rated Wrist Hand Evaluation (PRWHE) total scores

Treatment	Preoperative	One year postoperative	Improvement	P-value
All	65 (17)	30 (24)	-35 (24)	< 0.001
USO	68 (16)	34 (25)	-34 (21)	< 0.001
TFCC reinsertion	62 (18)	28 (22)	-34 (24)	< 0.001
Pisiformectomy	66 (15)	29 (25)	-37 (25)	< 0.001

*USO* ulnar shortening osteotomy, *TFCC* triangular fibrocartilage complex

**Fig. 3:**
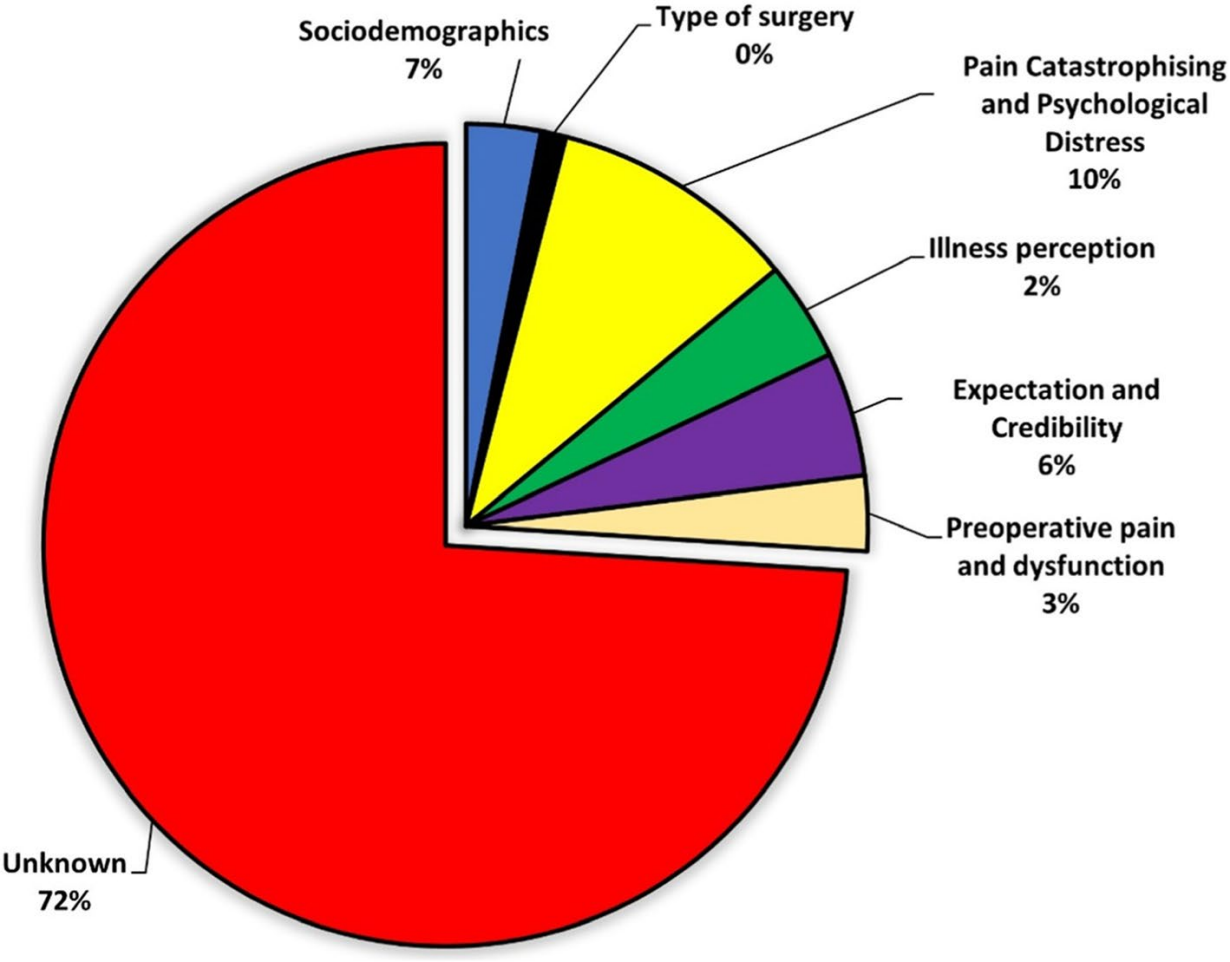
Pie chart on the increase in explained variance (R^2^) of 1-year postoperative pain and function measured with the Patient Rated Wrist/Hand Evaluation (PRWHE). Each slice represents the added R^2^ from a set of variables consecutively added to the linear regression models

The final model explained 26% of the variance in the 1-year postoperative PRWHE total score (Additional file 1). The consecutive relative contribution per set of variables is displayed in Fig. 3. Higher PCS score (B = 0.44; *β* = 0.19) and a higher preoperative PRWHE total score (B = 0.36; *β* = 0.25) were independently associated with a higher (worse) 1-year postoperative PRWHE total score. A higher CEQ Expectancy score (B = -1.63; *β* = -0.25) was independently associated with a lower (better) 1-year postoperative PRWHE total score (Table 2).

A posthoc analysis showed that a higher CEQ Expectancy score (B = -1.62; *β* = -0.25) and B-IPQ Consequence score (B = -2.15; *β* = -0.17) were independently associated with larger improvement in PRWHE score after one year (Table 2). All other variables did not influence the amount of improvement in this model.

There were no indications for multicollinearity in the models as Pearson correlation between psychosocial variables ranged from -0.21 to 0.57 (Additional file 3), and the VIF ranged from 1.05 to 2.15.

## Discussion

This study evaluated the impact of psychosocial factors on patient-reported pain and dysfunction both before and one year after surgery for ulnar-sided wrist pathology. Patients with more pain catastrophising behaviour or poor illness perception reported worse pain and dysfunction before surgery. Higher levels of pain catastrophising were associated with a worse 1-year outcome of surgery. In contrast, higher expectations of the treatment effect were associated with a better outcome. Higher expectations of treatment outcome increased the effectiveness of surgical treatment, while pain catastrophising and psychological distress did not seem to affect the effectiveness.

The first strength of our study is our routine longitudinal outcome management, including preoperative measurements, that allowed us to investigate the impact of psychosocial variables on treatment outcomes and change of scores. Second, we also evaluated the impact of illness perception and patients expectations besides the more broadly studied factors such as pain catastrophising and depression. Third, the large sample size allowed for multivariable testing of all psychosocial concepts simultaneously with adequate power.

This study also has limitations. First, it was impossible to determine a causal effect between psychosocial factors and patient-reported pain and function. While we have confirmed a strong association between the two, the effect can be bidirectional. For example, a more negative psychosocial profile may lead to worse pain or vice versa. Future research should determine the direction of the association between psychosocial factors and patient-reported pain and function. Second, we only included patients with ulnar-sided wrist pathology who were scheduled for surgical treatment. From previous research on thumb base osteoarthritis, we know that patients scheduled for surgical treatment have a worse psychosocial profile than their nonsurgically treated counterparts [37]. Therefore, the results of this study may not be generalisable to nonsurgically treated patients with wrist pathology. A third limitation was the missing data. Participation in the routine outcome measurement system is voluntary, and we have found that the response rate drops to approximately 50% after one year [18]. Nonrespons was more common in males, which is a known risk factor in routine outcome measurement of elective surgery [38]. We did not observe differences in the pain, function, and psychosocial scores between full and partial/non-responders. Therefore, we think that the missing data does not jeopardise the conclusions of our study. Fourth, we could not include the degree of anatomical abnormality in the models because there was no standardised workup protocol for patients with ulnar-sided wrist pain. For example, not all patients had a prior conventional x-ray, wrist arthroscopy, and MRI. Although previous research suggested that anatomical abnormalities were of limited value in explaining pain and dysfunction, incorporating them can better understand ulnar-sided wrist pain.

We showed that pain catastrophising behaviour and poorer illness perception were strongly associated with higher levels of patient-reported pain and dysfunction in patients with ulnar-sided wrist pathology. In our model, 35% of the explained variance could be attributed to pain catastrophising, anxiety and depression, and illness perception, which is in line with similar models from previous studies on carpal tunnel syndrome (20–25%) [13], Quervain’s tenosynovitis (27%) [12], and thumb base osteoarthritis (42%) [10]. In this study, higher levels of pain catastrophising were also independently associated with more pain and dysfunction one year after surgery. Teunis et al. also found poorer outcomes in patients with pain catastrophising behaviour after distal radius fracture surgery [39]. Another study found that pain catastrophising and anxiety and depression were associated with worse pain and function after three months of treatment in patients with degenerative wrist pathology (only 2% in their sample consisted of ulnar-sided wrist problems) [40]. In contrast to pain catastrophising, higher treatment expectations were independently associated with a better 1-year outcome. This is in line with a previous study on the surgical outcome of Quervain’s disease [33]. Interestingly, the mean improvement during the first year after surgery -although with a higher level of pain and dysfunction at intake- did not seem to differ based on pain catastrophising or anxiety and depression. A similar observation was reported by London et al. [40].

The relationship between psychosocial factors and pain and dysfunction in patients with ulnar-sided wrist pathology provides implications and recommendations for clinical practice. First, hand surgeons should be aware of the substantial relationship between patient-reported pain and dysfunction and psychosocial factors before surgery. A part of the complaints may stem from negative psychosocial factors, or the complaints may exhibit negative psychosocial factors. Second, operating patients with a more negative psychosocial profile report similar improvement than those with a more feasible psychosocial profile meaning that these should not be withheld from surgical treatment. However, considering the greater degree of complaints before surgery, patients with a more negative psychosocial profile before surgery still have worse 1-year treatment outcomes than patients with a more positive psychosocial profile. Therefore, hand surgeons should question whether patients with a more negative psychosocial profile are sufficiently treated with surgical interventions alone. For example, patients may benefit from counselling on psychosocial factors. This is encouraged by Zale et al., who concluded that patients benefit from learning resiliency skills [41]. Another study successfully changed illness perceptions using an intervention targeting patients’ perceptions of the consequences of myocardial infarction that resulted in accelerated patients’ return to work [42]. Similarly, an intervention aiming to improve patients’ psychosocial profile, such as illness perception in patients with ulnar-sided wrist pain, may improve treatment outcomes. Furthermore, we found that patients with higher outcome expectations had a greater improvement from surgical treatment. Thus, enhancing patient’s expectations even further might increase the effectiveness of surgery. As hand surgeons, we are not trained to treat symptoms of pain catastrophising, anxiety, depression, and illness perception. Therefore, a consultation with a mental health provider might be used alongside nonsurgical interventions before proceeding to more invasive treatment options for ulnar-sided wrist pain.

## Supplementary Information

**Additional file 1. **

**Additional file 2. **

**Additional file 3. **

**Additional file 4. **

## Data Availability

The datasets used and/or analysed during the current study are available from the corresponding author on reasonable request.

## Abbreviations

PCS: Pain catastrophising scale
PHQ: Patient health questionnaire
B-IPQ: Brief illness perception questionnaire
CEQ: Credibility expectancy questionnaire
PRWHE: Patient rated wrist/hand evaluation
SD: Standard deviation
IQR: Interquatile range
VIF: Variance inflation factor
USO: Ulna shortening osteotomy
TFCC: Triangular fibrocartilage complex
