# Supplementary material for: The impact of psychosocial variables on initial presentation and surgical outcome for ulnar-sided wrist pathology: a cohort study with 1-year follow-up
Source: BMC Musculoskelet Disord. 2022 Feb 1;23:109. doi: 10.1186/s12891-022-05045-x (PMC8808973; doi:10.1186/s12891-022-05045-x)
Supplement: Supplementary file 1 — Additional file 1. [file 12891_2022_5045_MOESM1_ESM.docx]

**Table s1:**Preoperative hierarchical linear regression model. The table shows the unstandardised coefficients (B), corresponding 95% confidence intervals, and standardised coefficients (β) for the associations between each variable and the Patient Rated Wrist Evaluation (PRWHE) total score.

|  | **Step 1**  **Sociodemographics** | | **Step 2**  **Planned surgery** | | **Step 3**  **Pain catastrophising + psychological distress** | | **Step 4**  **Illness perception** | |
| --- | --- | --- | --- | --- | --- | --- | --- | --- |
| **Variable** | **B [95%CI]** | **β** | **B [95%CI]** | **β** | **B [95%CI]** | **β** | **B [95%CI]** | **β** |
| SexFemales | 7.96 [ 4.13; 11.79]*** | 0,2 | 7.96 [ 4.14; 11.78]*** | 0,2 | 7.67 [ 4.11; 11.23]*** | 0,19 | 4.92 [ 1.79; 8.05]*** | 0,12 |
| Age (yrs.) | 0.13 [ 0.01; 0.24]* | 0,1 | 0.1 [ -0.02; 0.22] | 0,08 | 0.18 [ 0.07; 0.3]** | 0,15 | 0.18 [ 0.07; 0.28]** | 0,15 |
| Dominant side affected = No | 1.05 [ -2.23; 4.33] | 0,03 | 0.81 [ -2.48; 4.09] | 0,02 | 0.78 [ -2.28; 3.84] | 0,02 | 1.89 [ -0.78; 4.56] | 0,05 |
| Type of work = Light | 1.81 [ -2.89; 6.51] | 0,05 | 2.13 [ -2.6; 6.86] | 0,06 | 3.54 [ -0.88; 7.95] | 0,1 | 2.33 [ -1.51; 6.17] | 0,06 |
| Type of work = Medium | 3.84 [ -0.9; 8.58] | 0,1 | 4.03 [ -0.7; 8.76] | 0,11 | 4.98 [ 0.56; 9.39]* | 0,13 | 3.68 [ -0.18; 7.54] | 0,1 |
| Type of work = Heavy | 3.1 [ -2.49; 8.69] | 0,07 | 3.46 [ -2.13; 9.05] | 0,07 | 4.32 [ -0.9; 9.53] | 0,09 | 2.24 [ -2.32; 6.8] | 0,05 |
| Second opinion = No | -4.98 [ -10.01; 0.05] | -0,09 | -5.33 [ -10.39; -0.27]* | -0,1 | -6.17 [ -10.9; -1.44]* | -0,12 | -2.72 [ -6.93; 1.49] | -0,05 |
| Duration of symptoms (mos.) | -0.01 [ -0.07; 0.05] | -0,02 | -0.02 [ -0.09; 0.04] | -0,04 | -0.03 [ -0.09; 0.03] | -0,04 | 0 [ -0.05; 0.05] | 0 |
| Treatment = TFCC reinsertion |  |  | -4.44 [ -8.64; -0.24]* | -0,13 | -2.72 [ -6.65; 1.21] | -0,08 | -1.05 [ -4.49; 2.38] | -0,03 |
| Treatment = Pisiformectomy |  |  | -2.82 [ -7.39; 1.75] | -0,07 | -2.3 [ -6.55; 1.95] | -0,06 | -0.85 [ -4.56; 2.86] | -0,02 |
| PCS score |  |  |  |  | 0.5 [ 0.33; 0.67]*** | 0,29 | 0.19 [ 0.02; 0.36]* | 0,11 |
| PHQ score |  |  |  |  | 0.88 [ 0.22; 1.53]** | 0,13 | 0.3 [ -0.31; 0.9] | 0,04 |
| B-IPQ Consequences |  |  |  |  |  |  | 3.26 [ 2.36; 4.15]*** | 0,36 |
| B-IPQ Timeline |  |  |  |  |  |  | 0.09 [ -0.55; 0.73] | 0,01 |
| B-IPQ Personal Control |  |  |  |  |  |  | -0.23 [ -0.83; 0.37] | -0,03 |
| B-IPQ Identity |  |  |  |  |  |  | 1.88 [ 1.09; 2.67]*** | 0,23 |
| B-IPQ Concern |  |  |  |  |  |  | -0.62 [ -1.32; 0.08] | -0,09 |
| B-IPQ Understanding |  |  |  |  |  |  | 0.04 [ -0.67; 0.74] | 0 |
| B-IPQ Emotional Respons |  |  |  |  |  |  | 0.58 [ -0.09; 1.25] | 0,09 |
| R^2^ | 0.07 |  | 0.08 |  | 0.21 |  | 0.42 |  |
| Adjusted R^2^ | 0.05 |  | 0.06 |  | 0.18 |  | 0.39 |  |
| Sig. F-Change | <0.001 |  | 0.12 |  | <0.001 |  | <0.001 |  |

Abbreviations: B= unstandardized beta coefficient; β= standardized beta; CI= Confidence Interval; Ref= reference level; USO= ulnar shortening osteotomy; TFCC= Triangular Fibrocartilaginous Complex; PCS= Pain Catastrophizing Scale; PHQ= Patient Health Questionnaire; B-IPQ= Brief Illness Perception Questionnaire
*p <0.05; **p<0.01; ***p<0.001
