# Supplementary material for: The impact of psychosocial variables on initial presentation and surgical outcome for ulnar-sided wrist pathology: a cohort study with 1-year follow-up
Source: BMC Musculoskelet Disord. 2022 Feb 1;23:109. doi: 10.1186/s12891-022-05045-x (PMC8808973; doi:10.1186/s12891-022-05045-x)
Supplement: Supplementary file 2 — Additional file 2. [file 12891_2022_5045_MOESM2_ESM.docx]

**Table s2:** Postoperative hierarchical linear regression model. The table shows the unstandardised coefficients (B), corresponding 95% confidence intervals, and standardised coefficients (β) for the associations between each variable and the Patient Rated Wrist Evaluation (PRWHE) total score.

|  | **Step 1**  **Sociodemographics** | | **Step 2**  **Surgery** | | **Step 3**  **Pain catastrophising + Psychological distress** | | **Step 4**  **Illness perception** | | **Step 5**  **Expectations from treatment** | | **Step 6**  **Preop. Pain and dysfunction** | |
| --- | --- | --- | --- | --- | --- | --- | --- | --- | --- | --- | --- | --- |
| **Variable** | **B [95%CI]** | **β** | **B [95%CI]** | **β** | **B [95%CI]** | **β** | **B [95%CI]** | **β** | **B [95%CI]** | **β** | **B [95%CI]** | **β** |
| SexFemales | 2.83 [ -4.64; 10.3] | 0,05 | 3.05 [ -4.43; 10.53] | 0,05 | 1.86 [ -5.28; 9] | 0,03 | 0.31 [ -6.91; 7.52] | 0,01 | 0.45 [ -6.56; 7.45] | 0,01 | -1.46 [ -8.42; 5.49] | -0,02 |
| Age (yrs.) | -0.06 [ -0.28; 0.16] | -0,04 | -0.09 [ -0.33; 0.15] | -0,05 | 0.02 [ -0.21; 0.26] | 0,01 | 0.03 [ -0.21; 0.26] | 0,02 | 0.07 [ -0.16; 0.3] | 0,04 | 0.02 [ -0.21; 0.25] | 0,01 |
| Dominant side affected`No | 3.42 [ -2.62; 9.46] | 0,07 | 3.29 [ -2.75; 9.34] | 0,07 | 3.82 [ -1.93; 9.58] | 0,08 | 4.13 [ -1.65; 9.91] | 0,09 | 4.43 [ -1.18; 10.04] | 0,09 | 3.78 [ -1.72; 9.29] | 0,08 |
| Type of work = Light | -5.95 [ -14.52; 2.63] | -0,11 | -6.12 [ -14.74; 2.49] | -0,12 | -4.89 [ -13.1; 3.33] | -0,09 | -6.04 [ -14.29; 2.21] | -0,12 | -3.48 [ -11.59; 4.62] | -0,07 | -4.23 [ -12.18; 3.71] | -0,08 |
| Type of work = Medium | 0.38 [ -8.15; 8.9] | 0,01 | 0.2 [ -8.32; 8.72] | 0 | 0.81 [ -7.33; 8.94] | 0,02 | -0.66 [ -8.93; 7.6] | -0,01 | 1.51 [ -6.58; 9.6] | 0,03 | 0.83 [ -7.11; 8.76] | 0,02 |
| Type of work = Heavy | -6.92 [ -17.22; 3.38] | -0,11 | -6.85 [ -17.16; 3.46] | -0,11 | -4.95 [ -14.79; 4.89] | -0,08 | -6.06 [ -16.02; 3.89] | -0,09 | -4.99 [ -14.66; 4.68] | -0,08 | -5.29 [ -14.76; 4.18] | -0,08 |
| Second opinion = No | -3.13 [ -12.59; 6.34] | -0,04 | -3.62 [ -13.24; 6] | -0,05 | -4.18 [ -13.34; 4.98] | -0,05 | -4.07 [ -13.6; 5.46] | -0,05 | -3.68 [ -12.93; 5.56] | -0,05 | -2.36 [ -11.45; 6.73] | -0,03 |
| Duration of symptoms (mos.) | 0.13 [ -0.03; 0.3] | 0,1 | 0.11 [ -0.06; 0.28] | 0,08 | 0.09 [ -0.07; 0.25] | 0,07 | 0.09 [ -0.08; 0.26] | 0,07 | 0.07 [ -0.1; 0.23] | 0,05 | 0.06 [ -0.1; 0.22] | 0,05 |
| Treatment = TFCC reinsertion |  |  | -5.62 [ -13.28; 2.05] | -0,12 | -2.05 [ -9.46; 5.37] | -0,04 | -1.39 [ -8.82; 6.03] | -0,03 | 0.1 [ -7.15; 7.34] | 0 | 0.66 [ -6.44; 7.76] | 0,01 |
| Treatment = Pisiformectomy |  |  | -5.73 [ -13.99; 2.52] | -0,1 | -5.36 [ -13.22; 2.49] | -0,1 | -5.58 [ -13.43; 2.28] | -0,1 | -3.86 [ -11.53; 3.8] | -0,07 | -3.22 [ -10.74; 4.3] | -0,06 |
| PCS score |  |  |  | #N/A | 0.62 [ 0.3; 0.95]*** | 0,26 | 0.48 [ 0.1; 0.86]* | 0,2 | 0.5 [ 0.14; 0.87]** | 0,21 | 0.44 [ 0.08; 0.81]* | 0,19 |
| PHQ score |  |  |  | #N/A | 0.94 [ -0.27; 2.15] | 0,1 | 0.48 [ -0.85; 1.81] | 0,05 | 0.15 [ -1.15; 1.45] | 0,02 | -0.02 [ -1.29; 1.26] | 0 |
| B-IPQ Consequences |  |  |  | #N/A | #N/A | #N/A | 1.49 [ -0.48; 3.45] | 0,12 | 1.69 [ -0.22; 3.6] | 0,13 | 0.31 [ -1.73; 2.35] | 0,02 |
| B-IPQ Timeline |  |  |  | #N/A | #N/A | #N/A | 0.34 [ -1.12; 1.8] | 0,03 | -0.4 [ -1.86; 1.07] | -0,04 | -0.33 [ -1.77; 1.1] | -0,03 |
| B-IPQ Personal Control |  |  |  | #N/A | #N/A | #N/A | 0.36 [ -0.97; 1.69] | 0,03 | 0.14 [ -1.16; 1.44] | 0,01 | 0.27 [ -1; 1.55] | 0,02 |
| B-IPQ Identity |  |  |  | #N/A | #N/A | #N/A | 1.28 [ -0.53; 3.1] | 0,11 | 1.17 [ -0.59; 2.93] | 0,1 | 0.53 [ -1.23; 2.3] | 0,04 |
| B-IPQ Concern |  |  |  | #N/A | #N/A | #N/A | -1.14 [ -2.69; 0.41] | -0,12 | -1.33 [ -2.84; 0.18] | -0,14 | -1.19 [ -2.66; 0.29] | -0,12 |
| B-IPQ Understanding |  |  |  | #N/A | #N/A | #N/A | -0.24 [ -1.71; 1.24] | -0,02 | 0.2 [ -1.25; 1.64] | 0,02 | 0.19 [ -1.23; 1.61] | 0,02 |
| B-IPQ Emotional Respons |  |  |  | #N/A | #N/A | #N/A | 0.86 [ -0.62; 2.33] | 0,1 | 1.21 [ -0.22; 2.65] | 0,14 | 1.08 [ -0.33; 2.49] | 0,13 |
| CEQ Expectancy |  |  |  |  |  |  |  |  | -1.64 [ -2.45; -0.82]*** | -0.25 | -1.63 [ -2.43; -0.83]*** | -0.25 |
| Preop PRWHE total score |  |  |  |  |  |  |  |  |  |  | 0.36 [ 0.15; 0.57]*** | 0.25 |
| R^2^ | 0.03 |  | 0.04 |  | 0.14 |  | 0.18 |  | 0.23 |  | 0.26 |  |
| Adjusted R^2^ | 0.00 |  | 0.00 |  | 0.10 |  | 0.11 |  | 0.16 |  | 0.20 |  |
| Sig. F-Change | 0.41 |  | 0.26 |  | <0.001 |  | 0.19 |  | <0.001 |  | 0.001 |  |

Abbreviations: B= unstandardized beta coefficient; β= standardized beta; CI= Confidence Interval; Ref= reference level; USO= ulnar shortening osteotomy; TFCC= Triangular Fibrocartilaginous Complex; PCS= Pain Catastrophizing Scale; PHQ= Patient Health Questionnaire; B-IPQ= Brief Illness Perception Questionnaire
*p <0.05; **p<0.01; ***p<0.001
