# Supplementary material for: The impact of psychosocial variables on initial presentation and surgical outcome for ulnar-sided wrist pathology: a cohort study with 1-year follow-up
Source: BMC Musculoskelet Disord. 2022 Feb 1;23:109. doi: 10.1186/s12891-022-05045-x (PMC8808973; doi:10.1186/s12891-022-05045-x)
Supplement: Supplementary file 3 — Additional file 3. [file 12891_2022_5045_MOESM3_ESM.docx]

**Table S3:** Correlation matrix of psychosocial variables of all 423 patients.

| Variable | 1 | 2 | 3 | 4 | 5 | 6 | 7 | 8 | 9 | 10 |
| --- | --- | --- | --- | --- | --- | --- | --- | --- | --- | --- |
| 1 PCS score | 1 |  |  |  |  |  |  |  |  |  |
| 2 PHQ score | 0,45 | 1 |  |  |  |  |  |  |  |  |
| 3 B-IPQ Consequences | 0,36 | 0,30 | 1 |  |  |  |  |  |  |  |
| 4 B-IPQ Timeline | 0,31 | 0,13 | 0,24 | 1 |  |  |  |  |  |  |
| 5 B-IPQ Personal Control | -0,06 | 0,04 | -0,02 | 0,08 | 1 |  |  |  |  |  |
| 6 B-IPQ Treatment Control | -0,13 | -0,10 | -0,01 | -0,21 | -0,05 | 1 |  |  |  |  |
| 7 B-IPQ Identity | 0,29 | 0,19 | 0,51 | 0,30 | -0,03 | -0,03 | 1 |  |  |  |
| 8 B-IPQ Concern | 0,44 | 0,32 | 0,46 | 0,34 | 0,03 | -0,06 | 0,46 | 1 |  |  |
| 9 B-IPQ Understanding | -0,10 | -0,03 | 0,07 | -0,02 | 0,06 | 0,23 | 0,07 | -0,06 | 1 |  |
| 10 B-IPQ Emotional Respons | 0,52 | 0,51 | 0,48 | 0,26 | 0,05 | -0,06 | 0,42 | 0,57 | -0,03 | 1 |

Abbreviations: PCS= Pain Catastrophizing Scale= PHQ: Patient Health Questionnaire= B-IPQ: Brief Illness Perception Questionnaire
