# Supplementary material for: The impact of psychosocial variables on initial presentation and surgical outcome for ulnar-sided wrist pathology: a cohort study with 1-year follow-up
Source: BMC Musculoskelet Disord. 2022 Feb 1;23:109. doi: 10.1186/s12891-022-05045-x (PMC8808973; doi:10.1186/s12891-022-05045-x)
Supplement: Supplementary file 4 — Additional file 4. [file 12891_2022_5045_MOESM4_ESM.docx]

**The Hand Wrist Study Group**

Hand-Wrist Study Group members include: J.S. Teunissen^1,2^, M.J.W. van der Oest^2,3,4^, D.E. van Groeninghen^1^, R. Feitz^1,2^, S.E.R. Hovius^1,2^, E.P.A. Van der Heijden^1,5^, R.A.M. Blomme^2,6^, B.J.R. Sluijter^2,7^, D.J.J.C. van der Avoort^2,8^, A. Kroeze^2,9^, J. Smit^2^, J. Debeij^2,10^, E.T. Walbeehm^2,1^, G.M. van Couwelaar^2,12^, J.P. de Schipper^2,13^, J.F.M. Temming^2,14^, J.H. van Uchelen^2,15^, H.L. de Boer^2,14^, K.P. de Haas^2^, K. Harmsen^2^, O.T. Zöphel^2,12^, J.S. Souer^2^, R. Koch^2,10^, G.J. Halbesma^2,14^, T.M. Moojen^2^, G.M. Vermeulen^2^, X. Smit^2,3,15^, R. van Huis^17^, P.Y. Pennehouat^17^, K. Schoneveld^17^, Y.E. van Kooij^3,4,17^, J.J. Veltkamp^17^, A. Fink^17^, W.A. de Ridder^3,4,17^, J. Tsehaie^3,4^, R. Poelstra^3,4^, M.C. Jansen^3,4^, L. Hoogendam^3,4^, J.D. Dekker^2^, M. Jansen-Landheer^2^, M. ter Stege^2^, L. Duraku^18^, R.M. Wouters^3,4^, R.W. Selles^3,4^, H.P. Slijper^2,3,4^, J.M. Zuidam^3,15^, J.W. Colaris^19^.

^1^Radboud University Medical Centre, Radboud Institute for Health Sciences, Department of Plastic, Reconstructive and Hand Surgery, Geert Grooteplein Zuid 10, 6525 GA, Nijmegen, The Netherlands
^2^Hand and Wrist Centre, Xpert Clinics, Laarderhoogtweg 12, 1101 EA, Amsterdam, The Netherlands
^3^Department of Plastic, Reconstructive and Hand Surgery, Erasmus MC, University Medical Centre
Rotterdam, Doctor Molewaterplein 40, 3015 GD, Rotterdam, The Netherlands
^4^Department of Rehabilitation Medicine, Erasmus MC, University Medical Centre
Rotterdam, Doctor Molewaterplein 40, 3015 GD, Rotterdam, The Netherlands
^5^Department of Plastic, Reconstructive and Hand Surgery, Jeroen Bosch Ziekenhuis, Henri Dunantstraat 1, 5223 GZ, ‘s-Hertogenbosch, The Netherlands
^6^Department of Plastic, Reconstructive and Hand Surgery, Medisch Spectrum Twente, Koningstraat 1, 7512 KZ, Enschede, The Netherlands
^7^Department of Plastic, Reconstructive and Hand Surgery, Groene Hart Ziekenhuis, Bleulandweg 10, 2803 HH, Gouda, The Netherlands
^8^Department of Plastic, Reconstructive and Hand Surgery, Ikazia Ziekenhuis, Montessoriweg 1, 3083 AN, Rotterdam, The Netherlands
^9^Department of Plastic, Reconstructive and Hand Surgery, Sint Anna Ziekenhuis, Bogardeind 2, 5664 EH, Geldrop, The Netherlands
^10^Department of Plastic, Reconstructive and Hand Surgery, HagaZiekenhuis, Els Borst-Eilersplein 275, 2545 AA, Den Haag, The Netherlands

^12^Department of Plastic, Reconstructive and Hand Surgery, Ziekenhuisgroep Twente, Geerdinksweg 141, 7555 DL, Hengelo, The Netherlands
^13^Department of Plastic, Reconstructive and Hand Surgery, Máxima Medical Centre, Dominee Theodor Fliednerstraat 1, 5631 BM, Eindhoven, The Netherlands
^14^Department of Plastic, Reconstructive and Hand Surgery, Catharina Ziekenhuis Eindhoven, Michelangelolaan 2, 5623 EJ, Eindhoven, The Netherlands
^15^Department of Plastic, Reconstructive and Hand Surgery, Isala Zwolle, Dokter van Heesweg 2, 8025 AB, Zwolle, The Netherlands
^16^Department of Plastic, Reconstructive and Hand Surgery, Franciscus Gasthuis, Kleiweg 500, 3045 PM, Rotterdam, The Netherlands
^17^Centre for Hand Therapy, Wallesteinlaan 45, 3554 HM, Xpert Handtherapie, Utrecht, The Netherlands
^18^Department of Plastic, Reconstructive and Hand Surgery, Amsterdam University Medical Centre, Meibergdreef 9, 1105 AZ, Amsterdam, The Netherlands
^19^Department of Orthopaedics& Sports Medicine, Erasmus MC, University Medical Centre
Rotterdam, Doctor Molewaterplein 40, 3015 GD, Rotterdam, The Netherlands
